# The Book World of Medicine and Science

**Published:** 1902-05-10

**Authors:** 


### The Book World of Medicine and Science.

Tommy Cornstalk: Being Some Account of the Less Notable Features of the South African War from the Point of View of the Australian Ranks. By J. H. M. Abbott, late Corporal First Australian Horse. (London: Longmans, Green and Co. 1902. Price 5s. net.)

This is a very readable and interesting book, the scope of which is well described in its rather long title as given above. Coming, as we may now hope we are, within reasonable distance of the time when the settlement of South Africa will have to be seriously undertaken, it is pleasant to be told by one who is evidently well acquainted with the Australian bush, that most of the land in South Africa is at least as good as that in his own country, and better watered. In fact it would appear from the account here given that with careful cultivation the land of South Africa ought to be turned to very good account. Turning to the chapters describing the author's hospital experiences, which were derived from a residence of over two months in the hospitals at Pretoria and Bloemfontein, we find many criticisms which appear to be the honest outcome of personal observation. Speaking of the orderlies, the author compares those of the R A.M.C. somewhat unfavourably with the St. John's Ambulance men. The latter, he says, are volunteers and enthusiasts, and treat their patients with more kindness and gentleness than the regular orderlies, even if they are not so well trained and disciplined; but then, with the lordly contempt for low wages which is characteristic of the Australian, he says "one is almost ready to excuse many of the shortcomings of the much-abused orderlies by consideration of the fact that you cannot expect the services of a perfect being when you pay him something that comes to a little more than a shilling a day. If a good personnel is required, then it will have to be paid for; and you won't get 'much of a man' to do the kind of work the R.A.M.C. are called upon to do under five." He notices with astonishment the number of what he calls "hale beings" who were to be found in hospitals, but as he says it is not easy in such busy times to find proof of malingering, however much it may be suspected. "If a man steadily persists in having a pain in his chest, and be fairly consistent in his symptoms, you cannot without very great difficulty prove him a liar." Throughout the book there is constant evidence of a deeply-rooted antipathy to red tape, but however severe the author may be about systems he has many kind words for those by whom the work was done and for the energy by which the many difficulties met with in establishing the hospitals were overcome. The whole book is very well written. The fascination of the country evidently took hold of the author, and memories of the lonely, mournful, mysterious veld, and of the excitement of battle, were strong within him when he took up the pen. Thus we have a crisp outdoor swing of style that carries us through the pages with ever-increasing interest, an interest which only slackens a little when, with the obvious intention of giving everyone a good word, he says that the Boers are not so black as they are painted—which after all is not saying much.

Report on Leprosy and the Homeless Leper Asylum, Matunga, Bombay, 1890–1897. By N. H. Choksy M.D., late Medical Officer in charge of the Asylum. (Bombay: British India Printing Works. 1901.)

An interesting report, dealing in the first instance with the whole subject of leprosy, and in the second with the special experience of the Asylum at Matunga. So far as the latter is concerned it is to be remarked that the institution is not a hospital for the treatment of the disease, but rather an asylum, its work being carried on chiefly among that most wretched and unfortunate class the vagrant lepers. All the resources of the institution were from the first brought to bear upon teaching the unfortunate inmates habits of decency, inducing them to submit to discipline, to which from their vagrant mode of life, they were perfect strangers, and in "converting them from the squalid, filthy, and foul-smelling lepers of the streets into the clean, decent, and well-behaved inmates of the largest asylum in India." Thus, at first, but little scientific work could be done; but in time observations accumulated, and in this report they are recorded. Such as they are they go to show the enormous improvement which is produced even in the fully-developed disease by cleanliness and by improvement of the diet. Dr. Choksy says that good food and clothing will not only prolong a leper's life, but apparently stay the course of the disease, as has been observed in the majority of the cases at Matunga where, "under good regime and sanitary measures the patients improve wonderfully." Although this report is somewhat late in appearing, it is a very interesting document.

Official Guide to Llandudno and District. By Alec G. May. (Manchester: Henry Blacklock and Co.)

This little guide-book, which is issued by the Town Improvement Association, gives a good idea of the attractions of this pleasant watering-place. The illustrations show how picturesque are the surroundings, while the details given as to climate, drainage, water-supply, etc., are such as to indicate how suitable a residence it must prove for many valetudinarians, besides the crowd of pleasure seekers who are catered for in the summer months. It is interesting to note that Llandudno has had the foresight to provide for its water-supply by buying up a considerable area of gathering ground in the mountain district about 16 miles away, including a couple of lakes, and that this watershed does not contain a single habitation. The purity of the water-supply is thus assured. A very large amount of money has also been spent upon drainage. What becomes of the sewage at the outfall we are not told, but we are assured that "no town in the kingdom is better drained."

A Small-pox Insurance Diary. (Messrs. Charles Letts and Co., 3 Royal Exchange, E.C.)

Messrs. Charles Letts, the well-known makers of diaries, have made arrangements with the General Accident Assurance Corporation, to issue insurances against small-pox to medical men purchasing one of a variety of most convenient pocket cases which they publish. We admire the perspicuity of Messrs. Letts in selecting medical men to benefit by this advantageous arrangement, which can be entered into with no inconvenience, and at the small cost of one shilling, for which sum he in any case obtains a useful article. As a matter of fact, there are very few un-revaccinated medical practitioners, and in selecting their clientele Messrs. Letts have no doubt studied the small-pox returns, and realised that the number of doctors who contract the disease is infinitesimally small. The insurance provides £1 weekly for five weeks, or £100 at death.

